# Comparison of Zinc Oxide Nanoparticle Integration into Non-Woven Fabrics Using Different Functionalisation Methods for Prospective Application as Active Facemasks

**DOI:** 10.3390/polym15173499

**Published:** 2023-08-22

**Authors:** Tânia Ferreira, Ana Catarina Vale, Alexandra C. Pinto, Rita V. Costa, Vânia Pais, Diana Sousa, Fernanda Gomes, Graça Pinto, José Guilherme Dias, Inês P. Moreira, Carlos Mota, João Bessa, Joana C. Antunes, Mariana Henriques, Fernando Cunha, Raul Fangueiro

**Affiliations:** 1 Fibrenamics, Institute of Innovation on Fiber-Based Materials and Composites, University of Minho, 4800-058 Guimarães, Portugal; taniaferreira@fibrenamics.com (T.F.); catarinavale@fibrenamics.com (A.C.V.); ritacosta@fibrenamics.com (R.V.C.); vaniapais@fibrenamics.com (V.P.); ines.moreira@fibrenamics.com (I.P.M.); carlosmota@fibrenamics.com (C.M.); joaobessa@fibrenamics.com (J.B.); fernandocunha@fibrenamics.com (F.C.); rfangueiro@fibrenamics.com (R.F.); 2 Centre for Textile Science and Technology (2C2T), University of Minho, 4800-058 Guimarães, Portugal; a77645@alunos.uminho.pt; 3 CEB, Centre of Biological Engineering, LIBRO—Laboratório de Investigação em Biofilmes Rosário Oliveira, University of Minho, 4710-057 Braga, Portugal; dianasousa14@hotmail.com (D.S.); fernandaisabel@ceb.uminho.pt (F.G.); gracapinto@ceb.uminho.pt (G.P.); mcrh@deb.uminho.pt (M.H.); 4 LABBELS, Associate Laboratory, University of Minho, 4710-057 Braga, Portugal; 5 Poleva—Termoconformados, S.A. Rua da Estrada 1939, 4610-744 Felgueiras, Portugal; josedias@poleva.pt

**Keywords:** air filters, functionalisation, zinc oxide nanoparticles, antibacterial, antiviral, facemasks

## Abstract

The development of advanced facemasks stands out as a paramount priority in enhancing healthcare preparedness. In this work, different polypropylene non-woven fabrics (NWF) were characterised regarding their structural, physicochemical and comfort-related properties. The selected NWF for the intermediate layer was functionalised with zinc oxide nanoparticles (ZnO NPs) 0.3 and 1.2wt% using three different methods: electrospinning, dip-pad-dry and exhaustion. After the confirmation of ZnO NP content and distribution within the textile fibres by morphological and chemical analysis, the samples were evaluated regarding their antimicrobial properties. The functionalised fabrics obtained via dip-pad-dry unveiled the most promising data, with 0.017 ± 0.013wt% ZnO NPs being mostly located at the fibre’s surface and capable of total eradication of *Staphylococcus aureus* and *Escherichia coli* colonies within the tested 24 h (ISO 22196 standard), as well as significantly contributing (**** *p* < 0.0001) to the growth inhibition of the bacteriophage MS2, a surrogate of the SARS-CoV-2 virus (ISO 18184 standard). A three-layered structure was assembled and thermoformed to obtain facemasks combining the previously chosen NWF, and its resulting antimicrobial capacity, filtration efficiency and breathability (NP EN ISO 149) were assessed. The developed three-layered and multiscaled fibrous structures with antimicrobial capacities hold immense potential as active individual protection facemasks.

## 1. Introduction

All the occurring virus outbreaks—SARS-CoV coronavirus (2002), Swine influenza (2009), Mers-CoV (2012) and SARS-CoV-2 (2019)—are based on airborne transmission and are related to respiratory problems and pulmonary disorders [1,2]. Some relevant infectious agents are viruses, bacteria and fungi that are spread through any activities that generate small particles suspended in the air or droplets [3], especially in indoor environments [4]. In addition to social distancing, the use of facemasks has shown to be crucial to slow down rapid transmission by preventing inhalation and trapping airborne particles. Conventional air filters based on unitary polymer solutions or microscale fibres usually have low filtration efficiency for aerosols or airborne nanoparticles because of their large pores. The development of high-performance air filter membranes is thus essential, which can be achieved through multiscaled and multilayered membranes. For this purpose, research is focusing on the use of composites and electrospun nanofibrous membranes of polymeric blends [5,6,7]. Facemasks with nanofiber-based technologies became particularly relevant in 2020 during the latest pandemic as virus-size filtering membranes were indispensable in containing SARS-CoV-2 that has 60–140 nm of diameter [8].

Conventional facemasks are the perfect growth environment for microorganisms, due to the close contact with human skin, water retention from breathing and porosity—paradoxically working as nurseries for potential pathogens thereby increasing the risk of microbial infection for the user. To overcome these problems, the development of facemasks with inherent antimicrobial properties is urgently sought. This can be achieved by denaturing the infectious agent completely or rendering it inactive, using quaternary ammonium compounds (QACs) or inorganic materials as metal and metal oxide nanoparticles (NPs), as those containing gold, silver, copper, titanium and zinc as metallic elements [9,10]. Even though the exact antimicrobial mechanism of the referred active agents is not fully understood yet, it may involve the disruption of the cell wall or viral envelope due to the physical adsorption, electrostatic interactions, generation of reactive oxygen species (ROS) and ions release [11]. There have been several studies and developments on facemasks, including the functionalisation of surgical ones [12], as well as reviews on trends and future perspectives [13]. However, facemasks with innovative materials that can actively repel or degrade viruses and bacteria, while maintaining high breathability, are still not fully developed and commercialised [10]. Zinc oxide (ZnO) NPs are affordable, available, biocompatible, biodegradable, and have a hexagonal prism shape which increases surface roughness thereby enhancing microbial cell adhesion [14,15,16]. More so, ZnO NP’ UV protection, photocatalytic activity, antimicrobial, self-cleaning, energy-harvesting and biosafety features further support their use in facemasks. Zn-doped NPs are indeed capable of endowing a fabric with superhydrophobic properties that facilitate cleaning, among other functionalities, including microbicidal capacity [17]. Nevertheless, few works have been dedicated to the development of multifunctional facemasks modified with ZnO NPs [18,19,20].

Hence, in this work, a polypropylene-based non-woven fabric (NWF) made of multilayered composites—with spunbonded-meltblown-spunbonded (SMS) configuration—was selected as the filtering layer, since these are a common option for biological protection [17,21]. This NWF was then functionalised with ZnO NPs using three different methods—electrospinning, dip-pad-dry or exhaustion method—and thereafter tested for their antimicrobial efficacy. A multiscaled facemask was then assembled and thermoformed using the three layers—where the ZnO NP-functionalised was the one in the middle. An outer layer was added for shape-forming purposes, additionally acting as passive filter, while the inner layer was selected for comfort. The resulting facemasks were then assessed for their microbiological protection, breathability and filtering capacity.

The high antimicrobial activity of ZnO NPs is well-known, including against the SARS-CoV-2 virus, which makes these nanomaterials suitable candidates for integration in facemasks [22]. Recent findings indicate that in situ ZnO NPs synthesis is an innovative approach for the fabrication of effective antiviral facemasks, capable of neutralizing the SARS-CoV-2 virus, thereby upgrading the facemasks from simple physical barriers to active catalytic materials [23,24]. NPs deposition in plasma-treated nanofibrous membranes, or NPs co-extrusion with the polymeric phase has also been found [25,26], envisioning its use in facemasks via strategies like fortification of the nanoscaled mat with a multilayer structure [16]. In the aftermath of the recent COVID-19 pandemic, the widespread adoption of such active facemasks could have significantly enhanced overall healthcare practices. Yet, the tragic event was eye-opening for the need to bolster preparedness for future pandemics. Finally, industries involved in materials processing, painting and polishing processes stand to benefit immensely from the protective capabilities of these advanced facemasks, promoting a safer working environment for professionals in diverse fields beyond healthcare.

## 2. Materials and Methods

**Figure 1:**
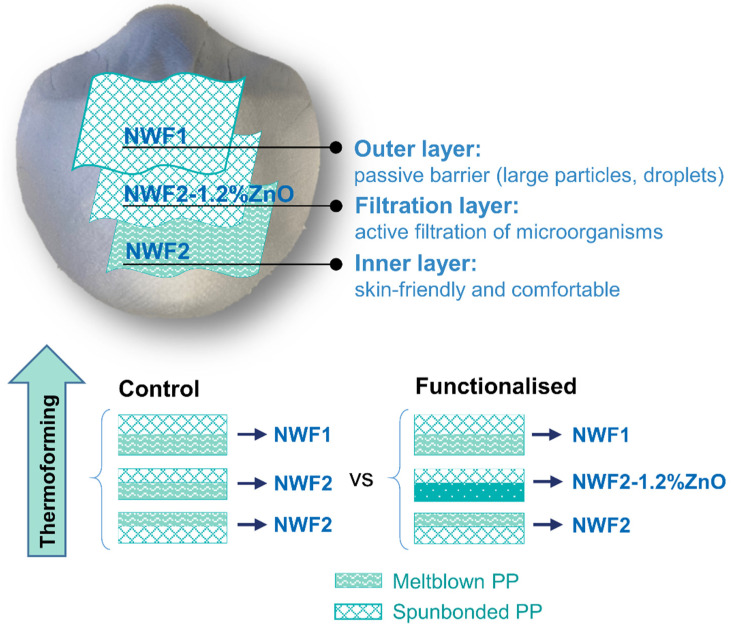
Schematic representation of the proposed three-layered facemask.

The present work aims at developing active and efficient antimicrobial facemasks, composed of three NWF layers following the SMS configuration, as illustrated by Figure 1. Two NWFs were studied, one denser and thicker than the other, and thus more adequate to function as a shape-forming outer layer. Each NWF consists of double-layered polypropylene (PP) fibrous substrates, one spunbonded (stiffer, more open structure) and another meltblown, deposited over the former, and being constituted by thinner fibres randomly disposed and forming a more compact, closed structure, and consequently more suitable to particle retention.

### 2.1. Materials

Two PP NWFs were purchased from Freudenberg, Germany. The raw materials polyamide, polyvinyl acetate, polyethylene glycol and zinc oxide nanoparticles (ZnO NPs) suspension (<100 nm particle size, ≤40 nm averaged particle size, 20wt% in H_2_O) were purchased to Sigma Aldrich, Burlington, MA, USA. Particle shape and size distribution were evaluated through transmission electron microscope (TEM) imaging (Figure S1) that were obtained using a Jeol JEM-2100-HT TEM (JEOL, Tokyo, Japan) operating at 200 kV and equipped with a fast-readout “OneView” 4k × 4k CCD camera that operates at 25 fps (300 fps with 512 × 512 pixel). Samples were sonicated for 30 s and then 5 μL of the solution was drop-casted on a carbon-coated 200 mesh copper grid (previously placed in parafilm). The drop dried for more than 2 h before TEM imaging. The median diameter was then deduced from TEM data measurements, simulating the diameter distribution with a log-normal function [27].

The solvents used were formic acid (FA) (98–100%, Fisher Scientific, Pittsburgh, PA, USA) and acetic acid (99.8%, Fluka, Charlotte, NC, USA).

To examine the antibacterial activity, Tryptic Soy Broth (TSB) and tryptic soy Agar (TSA) were next acquired from Liofilchem (Waltham, MA, USA), sodium chloride (NaCl) was obtained from J.T.Baker (Phillipsburg, NJ, USA), and polysorbate 80 (also known as tween 80) was obtained from VWR International (Radnor, PA, USA). The gram-positive bacterium *Staphylococcus aureus* (ATCC 6538, grown in TSB/TSA) was supplied by the American Type Culture Collection (ATCC, Manassas, VA, USA), and the gram-negative bacterium *Escherichia coli* (CECT 434, grown in TSB/TSA) was acquired from Colección Española de Cultivos Tipo (CECT, Paterna, Spain). For evaluating the antiviral activity, magnesium sulfate heptahydrate (MgSO_4_·7H_2_O) was acquired from CARLO ERBA Reagents S.A.S. (Emmendingen, Germany). Tris base was obtained from Fisher BioReagents (Pittsburgh, PA, USA). NaCl was also used. *E. coli* bacteriophage MS2 (ATCC 15597B1) and its respective host *E. coli* C-3000 (ATCC 15597) were acquired from ATCC. The host was grown in Luria-Bertani (LB) broth that was purchased from NZYTech (Lisboa, Portugal), and the bacteriophage was propagated using the double-layer methodology in LB agar (NZYTech) plates (1.2wt% agar) with a top layer of soft agar (0.6wt%) overnight at 37 °C.

### 2.2. Functionalisation with ZnO NPs

**Figure 2:**
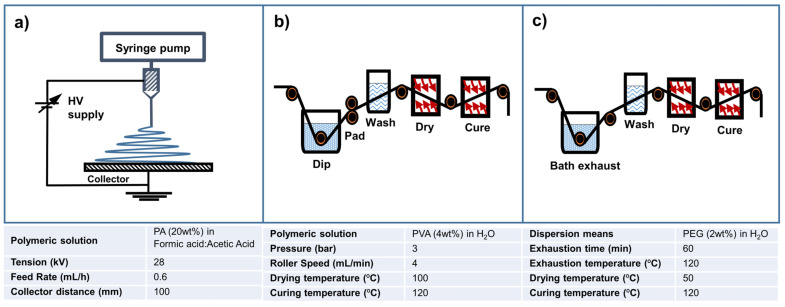
Methods for functionalisation of filtering layer with ZnO NPs: (**a**) electrospinning; (**b**) dip-pad-dry; and (**c**) exhaustion.

As Figure 2 summarises, three distinct functionalisation techniques were used to incorporate two low ZnO NPs concentrations (0.3 and 1.2wt%) in the intermediate layer of NWF 2 (NWF2).

For the electrospinning technique, the substrate was placed over a flat collector for the deposition of electrospun polyamide (PA) nanofibres, following the optimised electrospinning conditions reported [7,28], but this 20wt% PA in formic acid (90% *v*/*v*) and acetic acid (10% *v*/*v*) solution were here doped with 0.3 and 1.2wt% ZnO NPs. The injection syringe was filled with the solution with a fixed voltage of 28 kV applied to the steel capillary needle (22G) with 0.41 mm. The feeding rate was 0.6 mL/h and the needle-to-collector distance was 10 cm. Temperature and relative humidity were kept constant at 25 °C and 58%, respectively.

For the dip-pad-dry technique, a 4wt% polyvinyl alcohol (PVA) in distilled water solution was prepared overnight with the ZnO NPs, followed by sonication. The substrate was dipped in this solution for 15 s each side and then passed five times on the rolls working at a pressure of 3 bar and 4 m/min. After that, the samples were dried at 50 °C for 1 h and cured at 120 °C for 3 min.

For the exhaustion technique, a 2wt% polyethylene glycol (PEG) solution in distilled water containing the ZnO NPs was prepared overnight, also followed by sonication. The substrate was dipped in this solution and let to exhaustion for 1 h at 120 °C, followed by drying at 50 °C for 1 h and curing at 120 °C for 3 min.

### 2.3. Non-Woven Fabric Characterisation

The NWF comprehensive characterisation was carried out regarding their structural, physical, mechanical and thermal management properties.

For the structural properties, thickness and areal mass density were determined.

Thickness measurements were conducted on 11 mm diameter samples of each type of film using a handheld analogical micrometre with a dial indicator from Mitotoyo with a resolution of 0.01 mm, 10 mm pressing area, and 18 Pa of pressure. Ten samples/conditions were examined. The areal mass density was determined based on the ISO 3801 standard [29], which requires the samples to be conditioned in a standardized atmosphere of 20 °C with a relative humidity of 65%. The samples are cut with a minimum area of 100 cm^2^, conditioned, and then weighed. The results are presented in g/m^2^. For this test, at least five replicates were performed.

The thermoregulation and moisture management properties were determined using a moisture management tester (MMT equipment, model M290, SDLAtlas, Rock Hill, SC, USA). According to the AATCC Test Method 195-2012 [30], five samples (8 × 8 cm) were prepared and pre-conditioned in a controlled atmosphere and samples were evaluated by placing each sample between two horizontal electrical sensors.

The Alambeta measuring device was used to determine the transient and steady-state thermo-physiological properties, simulating in a very small initial contact the heat flux between skin and fabrics.

The flexibility and subsequent stiffness of the samples were evaluated with the following measurements of 20 cm by 2.5 cm, cut in the direction of the warp and weft. Hence, samples were tested with an apparatus enabling the determination of the bending stiffness (G). The results are presented as an average and standard deviation of five measurements. In the end, with the recorded values, it was possible to calculate the bending stiffness for the web and for the plot.

(1) $$G=0.10\;MC^{3}$$

where: G—bending stiffness (mg cm); M—mass per surface unit of the sample (g/m^2^); C—averaged length (of the five samples) required for the fall (cm).

The friction coefficient was determined using Frictorq equipment, allowing for the extraction of the static and kinetic coefficients.

The water contact angle was determined using an OCA 200 Data Physics apparatus (Filderstadt, Germany) connected to a video-based drop shape analyser OCA 15 plus software (version 1.2), following the standard ASTM-D7334-08 [31]. The average of 10 measurements is presented.

### 2.4. Functionalised Sample Characterisation

#### 2.4.1. SEM-EDS

Morphological analyses were performed in an Ultra-high-resolution Field Emission Gun Scanning Electron Microscopy (FEG-SEM), NOVA 200 Nano SEM, FEI Company, Hillsboro, OR, USA. Topographic images were obtained with a secondary electron detector at an acceleration voltage of 10 kV. Before morphological analyses, samples were covered with a thin film (35 nm) of Au-Pd (80–20 weight %) in a high-resolution sputter coater 208HR coupled to a MTM-20 Cressington High Resolution Thickness Controller (Cressington Company, Watford, UK). Chemical analyses of samples were performed with the Energy Dispersive Spectroscopy (EDS) technique, using an EDAX Si(Li) detector (Pleasanton, CA, USA) at an acceleration voltage of 15 kV.

#### 2.4.2. TEM

Morphological analyses of the functionalised samples plus control were performed using a protocol adapted from the work of Mast et al. [32]. Briefly, the samples were impregnated with Epon812 resin for 2 h and then placed in a mould filled with fresh Epon812 resin and cured for 48 h at 60 °C in an oven. After inclusion, with trimming of the specimen block, ultra-thin longitudinal sections with a thickness of 90 nm were obtained using the PowerTome PC Ultramicrotome (Boeckeler Instruments, Inc., Tucson, AZ, USA). Sections were placed on carbon and formvar coated copper grids (200 mesh). Electron micrographs of the samples were taken with a Jeol JEM-2100-HT TEM (JEOL, Tokyo, Japan) operating at 200 kV and equipped with a fast-readout “OneView” 4k × 4k CCD camera that operates at 25 fps (300 fps with 512 × 512 pixel).

#### 2.4.3. Attenuated Total Reflectance-Fourier Transform Infrared Spectroscopy (ATR-FTIR)

The presence of ZnO NPs was analysed by ATR-FTIR analysis using an IRAffinity-1S SHIMADZU Equipment (Kyoto, Japan). Each spectrum was obtained in transmittance mode using a diamond ATR crystal cell by the accumulation of 45 scans with a resolution of 4 cm^−1^ from 400 to 4000 cm^−1^, being acquired three spectra per condition.

#### 2.4.4. Ground State Diffuse Reflectance (GSDR)

Ground State Diffuse Reflectance (GSDR) was performed to compare the absorption spectrum of the non-woven fabric two without and with the ZnO NPs, at distinctive concentrations, incorporated following the different methods. The spectra were recorded in the 200 to 800 nm wavelength range, using a Shimadzu UV 2501PC Spectrophotometer (Shimadzu Corporation, Kyoto, Japan). Each sample was analysed in three different places to ensure a reliable analysis. The remission function (F(R)) was calculated according to the Kubelka-Munk equation:

(2) $$F\left(R\right)=\frac{{\left(1-R\right)}^{2}}{2R}=\frac{K}{S},$$

where K represents the absorption coefficient, S is the dispersion coefficient, and R is the reflectance.

#### 2.4.5. UV-Visible Spectrophotometry (UV-Vis)

Firstly, calibration curves relating to ZnO NPs concentration in water (with absorbance value ~375 nm (due to the band-gap absorption for ZnO resulting from removed electrons from the valence band to the conduction band [33,34,35]) were constructed, following a range of dilutions between 1:500 and 1:8000 that enabled reliable detection of the spectra’s region of interest. Results were plotted as absorbance vs. concentration (Figure S2). Then, absorbance scans of each sample (representative rectangles with 6 × 2 cm^2^) were collected between 190–1100 nm (resolution of 1 nm), with a UV-2600 UV-vis spectrophotometer by resorting to an integrating sphere (ISR-2600Plus) with a film holder for transmittance analysis (Shimadzu Corporation, Kyoto, Japan). The quantity of ZnO NPs loaded onto each functionalized NWF was estimated in an indirect manner. For each spectrum, absorbance peak height (values between 360–390 nm) was registered. The estimation of detected ZnO NPs presence (wt%) in these samples was then calculated taking into consideration detected ZnO NPs concentration (µg/mL) by means of the predetermined calibration curve, along with sample volume crossed by the laser that resulted from the determination of laser cross-section in transmission and sample thickness.

#### 2.4.6. Thermogravimetric Analysis (TGA)

The TGA analysis was performed using a SDT-2960 (TA Instruments) over a temperature range of 25–500 °C with a heating rate of 10 °C/min in an argon gas environment.

Thermal gravimetric analysis (TGA) measurements were conducted on an SDT-2960 from NETZSCH Q500 using a platinum pan. The TGA trace was obtained in the range of 25–500 °C under a nitrogen atmosphere, a flow rate of 200 mL/min, and a temperature rise of 10 °C/min. Results were plotted as the percentage of weight loss vs. temperature.

#### 2.4.7. Differential Scanning Calorimetry (DSC)

Differential scanning calorimeter (DSC) data were acquired on a Power Compensation Diamond DSC (Perkin Elmer, Waltham, MA, USA) with an Intracooler ILP, based on the standards ISO 11357-1:1997 [36], ISO 11357-2:1999 [37], and ISO 11357-3:1999 [38]. Tests were conducted under a nitrogen atmosphere with a flow rate of 200 mL/min and a heating rate of 10 °C/min. The thermogram was obtained in the range of 25–500 °C. Results were plotted as heat flow vs. temperature.

#### 2.4.8. Air and Water Vapour Permeability

The air permeability was performed using an air permeability tester TEXTEXT FX 3300-III, following the procedure established in the standard NP EN ISO 9237 [39]. A pressure of 100 Pa was used in a surface area of 20 cm^2^, with the air flux in the direction from the exterior to the interior. At least 10 measurements were performed for each sample, and the average and standard deviation were determined (L/m^2^·s).

The water vapour permeability (WVP) was performed using a water vapour permeability tester (TF165, TESTEX, Guangdong, China) and following the standard BS 7209:1990 [40]. The measurements were performed for 5 h, under a controlled environment (temperature of 20 °C and relative humidity of 65%) and after 1 h of sample stabilisation. The external side was placed facing the recipient interior so that the vapour flux was evaluated from the exterior to the interior. Three replicas were used for each sample so that the average and standard deviation were determined. Hence, the water vapour permeability was determined following the equation:

$$WVP(g/m^{2}/\mathrm{day})=\frac{24\times m}{A\times t}$$

where m was the lost mass (g); A was the sample exposed area (~0.0054113 m^2^); and t was the time between weighing (t = 5 h). And finally, the water vapour permeability index was determined based on the normalisation of the sample water vapour permeability (WVP_sample_) and standard water vapour permeability (WVP_standard_):

$$I\left(\%\right)=\frac{{WVP}_{sample}}{{WVP}_{standard}}\times 100$$

#### 2.4.9. Antimicrobial Activity

##### Antibacterial Activity

The antibacterial activity was tested against a Gram-positive (*Staphylococcus aureus* ATCC 6538) and a Gram-negative (*Escherichia coli* CECT 434) bacteria following the standard ISO 22196 [41]. The quantitative determination of antibacterial activity was carried out as follows. An inoculum was prepared in 20 ± 0.1 mL of TSB and incubated overnight at 37 °C and 120 rpm. The bacteria concentration was adjusted to 6 × 10^5^ cells/mL by absorbance reading and using the respective calibration curves. An amount of 400 μL from the previous suspension was added to each sample (3 × 3 cm) previously sterilized by ultraviolet radiation, and the test inoculum was covered with a piece of polyethylene film (2 × 2 cm). After an incubation period of 24 h at 37 °C, 10 mL of TSB + Tween 80 (TSB and 7.0 g of nonionic surfactant Tween 80 per liter) was added, and the samples were vortexed for 25 s. To achieve the number of living bacteria, a serial dilution plate count method was performed.

The assessment of the antibacterial activity of the samples was performed according to the standard ISO 22196—Measurement of antibacterial activity on plastics and other non-porous surfaces [41]. The antibacterial activity was evaluated against two strands: *Staphylococcus aureus* ATCC 6538 (Gram+) and *Escherichia coli* CECT 434 (Gram−). As a control, samples without the active agent ZnO NPs were used, to allow the determination of the antimicrobial activity (R):

$$R=U_{t}-A_{t}$$

where

$U_{t}$ is the average of the logarithm of the number of viable bacteria (N), in cells/cm^2^, taken from the control samples after 24 h;

$A_{t}$ is the average of the logarithm of the number of viable bacteria (N) in cells/cm^2^, taken from the functionalized samples after 24 h.

It is noteworthy that the ISO 22196:2007 standard did not specify the requirements for effective antibacterial activity. However, based on information from JIS Z 2801:2000 [42]—“Antimicrobial products—Test for antimicrobial activity and efficacy”, it was considered that a material exhibits effective antibacterial activity when the R-value, which represents antibacterial activity, is equal to or greater than 2. All assays were performed in duplicate and repeated at least three times.

##### Antiviral Activity

The antiviral activity of samples was tested following the methodology described in ISO 18184:2014—Textiles—Determination of antiviral activity of textile products, using bacteriophage MS2, commonly applied as a surrogate for pathogenic respiratory viruses [43]. The viral suspensions were prepared according to the methodology of ISO 18184 and were kindly provided by the Bacteriophage Biotechnology Group of the Center for Biological Engineering at University of Minho. Each sample (3 × 3 cm), sterilized by ultraviolet radiation, was inoculated with 400 μL of the viral suspension, prepared to a concentration of approximately 1 × 10^5^ Plaque Forming Units (PFU) per mL, and covered with a piece of polyethylene film (2 × 2 cm). After an incubation period of 24 h at 25 °C, 10 mL of Salt Mg buffer (SM buffer: 5.8 g NaCl, 2.0 g MgSO_4_7H_2_O and 50 mL 1 M TrisHCL pH 7.5 per liter) was added to the samples and mixed by vortex for 25 s. The wash-out suspension was diluted (10-fold serial dilution in SM buffer) and plated using the double agar overlayer method. Briefly, 100 µL of *Escherichia coli* ATCC 15597, previously grown for 18 h at 37 °C, were added to 5 mL of TSB soft agar (TSB containing 0.6% agar). The agar overlay was poured onto base plates containing TSA and swirled to allow uniform spread. An amount of 10 µL of the phage suspensions was plated on top of the soft agar, allowed to dry, and incubated for 18 h at 37 °C. Isolated lysed zones (plaques) indicating the presence of phage were counted. The antiviral activity (Mv) after 24 h incubation was evaluated using the following formula:

$$Mv=\log\left(Vb\right)-\log\left(Vc\right)$$

where

Mv is the antiviral activity value;

$\log\left(Vb\right)$ is the average logarithm of infectious titles after 24 h contact with the standard sample;

$\log\left(Vc\right)$ is the average logarithm of infectious titles after 24 h contact with the functionalised sample. The assays were performed in duplicate and replicated at least three times.

#### 2.4.10. Filtration Performance and Breathability

The breathability was performed using a GESTER equipment (GESTER (Quanzhou Gester International Co, Quanzhou, China), following the procedure established in the standard NP EN ISO 149 [44]. Hence, a maximum pressure of 2.4 mbar for inhalation at 95 L/min was used, and 3 mbar for exhalation at 160 L/min. For inhalation, the air flux was set from the exterior to the interior, and in the opposite direction for exhalation.

The filtration performance was tested through the penetration of sodium chloride aerosol, using a PFE Tester Auto Particle Filter Efficiency GT-RA09 from GESTER as well, by applying a flow rate of 95 L/min, and the maximum value was registered after 3.5 min of exposure. 

#### 2.4.11. Statistical Analysis

Distinct results were statistically analysed using the Prism software package (version 8.0.1; GraphPad Software). One-way analysis of variance (ANOVA) was performed, and means were compared by applying Tukey’s multiple-comparison test and Kruskal-Wallis as well for multi-comparison analyses. The statistical analyses performed were considered significant when *p* < 0.05.

## 3. Results and Discussion

### 3.1. NWF Characterisation

Both NWF1 and NWF2 were characterised concerning structural, physical and mechanical parameters, as well as their capacity for thermal and moisture management. The results obtained (Table 1) assisted in layer definition for the multilayer buildup of the facemask filtration system.

The studied structural parameters indicate that the NWF1 is thicker than the NWF2, which presents an undoubtedly suitable thickness for the intended application [45,46,47]. NWF1 is additionally endowed with a denser fibrous structure. This fact, combined with its higher bending stiffness, makes NWF1 more adequate to constitute the outer layer of the facemask, contributing to higher maintenance of the created tridimensional shape. In parallel, NWF2 was the substrate chosen for the intermediate filtering layer, with a lower stiffness facilitating the functionalisation processes.

Bending stiffness is a fabric property that could negatively affect the comfort sensation of the facemask user [48]. Equally important to mention are its equivalent static and kinetic friction coefficient, as well as its wettability. The friction coefficient is an important parameter associated with certain surface characteristics [49] like surface roughness. It can be used as a comfort value since it provides information related to tactile perception of the fabric under study. In this sense, low friction coefficients could offer better comfort properties, being linked to pleasant skin reactions to a direct contact with it. Wettability is also often influenced by surface topography, along with material composition and surface energy. A surface is hydrophilic if the contact angle is in the range of 0° ≤ θ ≤ 90°, hydrophobic if in the range 90° ≤ θ ≤ 150° and superhydrophobic if the contact angles are above 150°. Both NWFs under study are hydrophobic. This fact was somewhat expected, given that PP is a hydrophobic polymer [50] and, traditionally, NWF-based facemasks are water-repellent [10,46,51]. Taking into consideration that SARS-CoV-2 is primarily transmitted by respiratory droplets and aerosol and contact routes, a non-fouling surface is desired [17].

In what concerns thermal and moisture management properties, both NWFs were deemed appropriate to act as inner layer, with excellent OWTC values > 400% and very good OMMC values (0.6–0.8), according to the American Association of Textile Chemists and Colorists (AATCC) standard [52], with the NWF2 presenting slightly better values. As described by the manufacturer, OWTC is the difference in the cumulative moisture content between the two surfaces of the fabric in the unit testing time. In its turn, OMMC is an index to indicate the overall ability of the fabric to manage the transport of liquid moisture, which includes three aspects of performance: spreading speed hence drying speed, the moisture absorption rate of the bottom side and one-way liquid transportability. Higher overall moisture management capacity indicates better overall moisture transportability of the fabric. NWF2 does seem to be capable of transporting sweat and breathing metabolites from the skin-fabric microenvironment toward the exterior.

From the thermal properties characterized by the Alambeta, there are slight differences between NWFs that could be related to other fabric characteristics, such as structural ones. Since NWF1 is thicker and denser than NWF2, that may also have led to higher thermal resistance, accordingly to some previous studies that support the strong association between these parameters [53].

For these reasons, the NWF2 was chosen for the inner layer in contact with the skin.

### 3.2. Functionalised NWF Characterization

#### 3.2.1. Morphological and Chemical Analyses

**Figure 3:**
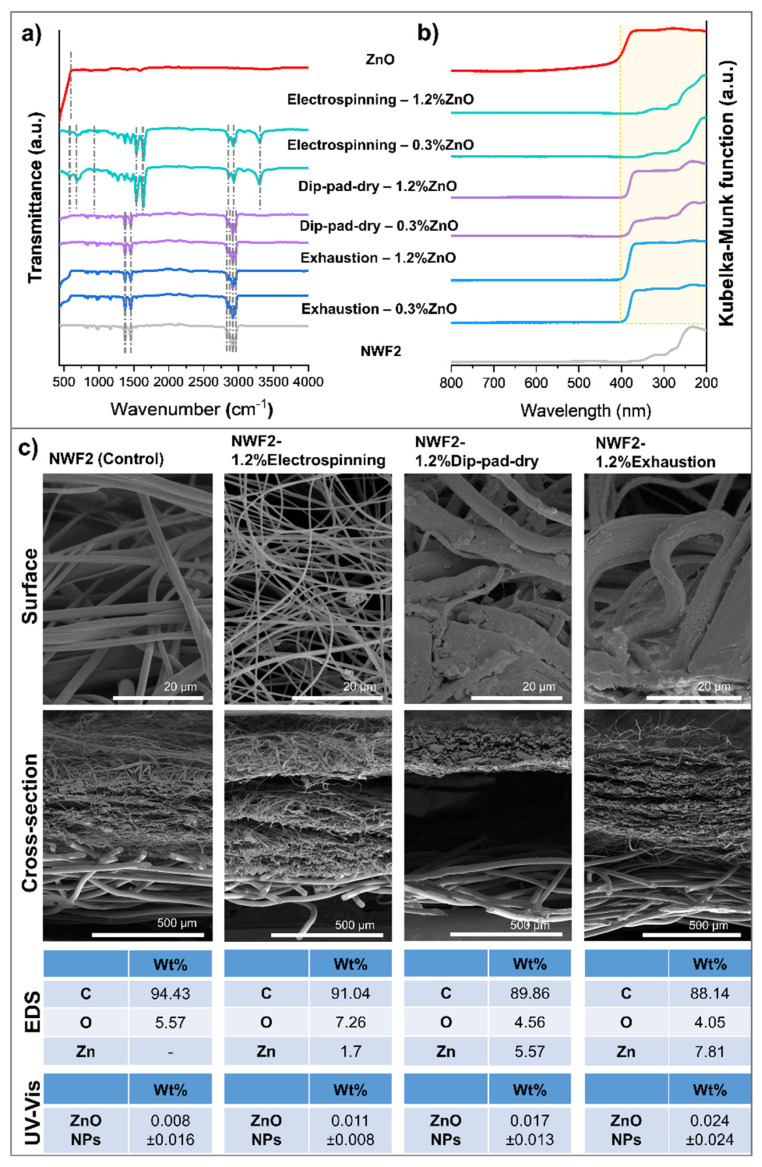
Physicochemical characterization for the confirmation of ZnO NPs incorporation: (**a**) ATR-FTIR spectra of the control and functionalised samples, with dashed lines highlighting main peaks of interest; (**b**) UV-Visible GSDR spectra of the control and functionalised samples; and (**c**) FEG-SEM surface and cross-section images of the NWF2 control and functionalised samples, and corresponding quantitative analyses using EDS and UV-vis.

The successful loading of ZnO NPs into NWF2 during textile functionalisation, using three different methodologies (electrospinning, dip-pad-dry and exhaustion) in two different concentrations (0.3 and 1.2wt%) was confirmed by ATR-FTIR and UV-visible spectroscopy. The ATR-FTIR spectra of all the functionalised samples follow a similar pattern to the NWF2 control (Figure 3a), with a new peak emerging near 500 cm^−1^ which is present in the control analysis of ZnO NPs. Particularly, the O–H bending vibration band arises at 625 and 620 cm^−1^, as well as the 563 and 465 cm^−1^ peaks that are attributed to the stretching vibration of Zn–O bonds [54]. This proves the presence of ZnO NPs in all functionalised samples, with the most intense ones appearing for the exhaustion method. It is also worthy to mention that distinct differences appeared in the spectra of the electrospun samples in comparison to the others. The former displays extra peaks at the amide I band, near 3300 cm^−1^, which can be explained by the N–H stretching vibrations of the polyamide nanofibres. Moreover, the asymmetric and symmetric stretching of CH_2_ appears at 2931 and 2861 cm^−1^, respectively. Amide bands of the polyamide appear at 1643 and 1532 cm^−1^, corresponding to the CO–CH symmetric bending vibration when combined with CH_2_ twisting. The peaks at 933 and 688 cm^−1^ are typically attributed to the stretching and bending vibrations of C–C bonds, while the band at 583 cm^−1^ may result from O=C–N bending [55]. Regardless, characteristic peaks of non-crystalline PP have been identified in all NWF-based spectra, namely of the CH_2_– group (2914 cm^−1^, 2841 cm^−1^) and the –CH_3_ group (2959 cm^−1^, 2871 cm^−1^, 1455 cm^−1^, 1378 cm^−1^) [56].

In turn, the GSDR spectrum of the ZnO NPs control presents a peak around 375 nm (Figure 3b), accordingly to some studies in the literature [33,34,35]. This peak was additionally visible in the functionalised samples, except the ones where ZnO NPs were added via electrospinning. This could indicate that the ZnO NPs incorporated within the nanofibres were not present in detectable amounts, or that it is heterogeneously distributed in the produced mats, so that the laser may, or may not, encounter NPs or NPs clusters during its crossing through the sample. The spectra observed for the samples prepared by electrospinning are indeed non-functionalised like the NWF2 spectrum. ZnO NPs-related peak appears as more intense in samples functionalised by the exhaustion method (26.87) than with dip-pad-dry (18.57), especially with the 1.2wt% ZnO NPs, which corroborates FTIR findings.

**Figure 4:**
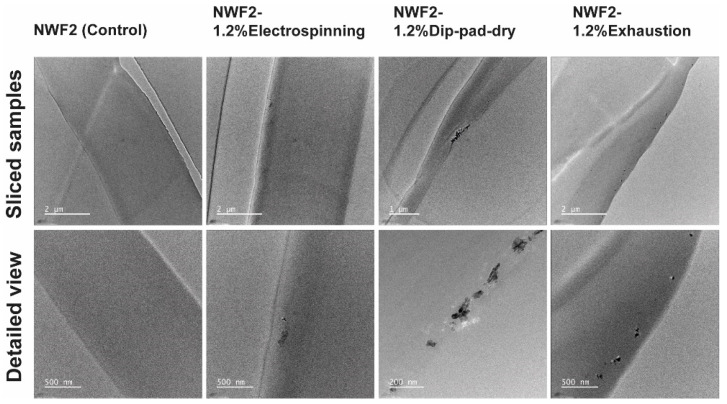
TEM images of longitudinal sections of the NWF2 control and functionalised samples.

From the FEG-SEM analysis (Figure 3c), it is possible to infer the different morphological aspects of the samples obtained following the three functionalisation methods. The electrospinning technique deposited a mat of randomly oriented PA nanofibres, following the team’s expertise [7] with ZnO NPs located within PA nanofiber extruding dispersion and deposited over the NWF2, whereas the other techniques placed the NPs onto/within the PP fibres of the NWF2. TEM observations of longitudinal sliced samples (Figure 4) unveiled a scarce presence of NPs within the PA fibres. The applied pressure in the padding step of the dip-pad-dry method has shortened (thickness of 0.36 ± 0.01 mm in comparison to 0.39 ± 0.02 mm of the control) the meltblown architecture creating compacted NWF, even after the optimisation of the wet pickup allowed by the equipment. Apparently, the meltblown-spunbonded interface was even weakened by this process, given that the vacuum created for SEM image acquisition created a visible void (black middle layer) between them. Either way, the NPs have been encrusted at the surface of the NWF2 microfibres, a fact that has been confirmed via TEM. Many NPs agglomerates are distinguishable, even after optimizing the dispersant media (preliminary experiments used PEG as dispersant media, following previously developed work of the team [57]), while taking profit from hydrogen bonds promoted by PVA and causing NP’s steric repulsion [58,59,60]. Finally, the exhaustion method produced a more homogeneous incorporation of the ZnO NPs over/within the whole sample, which may be due to the formulation used, the prolonged stirring bath, and the selected bath temperature. The representative EDS analysis confirmed the presence of a small quantity of the element Zn, caused by the functionalisation with ZnO NPs, with the results supporting once more that the exhaustion method collected the largest atomic percentage of Zn. EDS spectra (Figure S3) and UV-vis measurements (Figure 3) corroborated the later findings. TEM imaging also gave that impression, further enabling the visualisation of NPs, with some being located at the fibre’s surface, but many further into its core.

#### 3.2.2. Biological Studies

**Figure 5:**
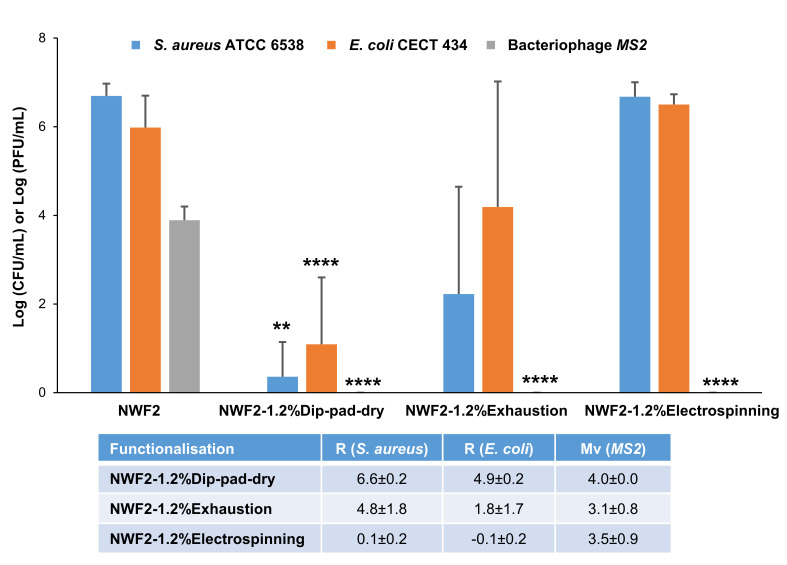
Antimicrobial activity of NWF2 and functionalised samples through the three tested methodologies against *Staphylococcus aureus* ATCC 6538, *Escherichia coli* CECT 434 and bacteriophage MS2, and their respective antibacterial activity (R) and antiviral activity (Mv) values. ** *p* < 0.01, **** *p* < 0.0001 compared with the respective positive control (without functionalisation).

The antimicrobial activity of functionalised NWF and respective control was evaluated against standard strains of the Gram-positive bacteria *Staphylococcus aureus* and the Gram-negative bacteria *Escherichia coli*. The number of microbial viable colonies after 24 h of incubation in liquid media under dynamic conditions was emphasized. Data from Figure 5 show that functionalized NWF could invoke a higher antimicrobial activity than the control (NWF as received), as expected. No statistically significant difference was observed between Gram-positive and Gram-negative cells, despite the complexity of the Gram-negative bacterial cell wall [61]. While comparing the three functionalisation methods with the control, dip-pad-dry showed to be the most effective and the only one that evidenced a significant log reduction on both bacteria tested (*p* < 0.05), reinforced by the corresponding R values. Based on the criterion of the consulted standards, it was considered that there was an antibacterial effect to R ≥ 2. Thus, NWF2 functionalized by dip-pad-dry showed antibacterial activity (R = 6.6 and 4.9, for *S. aureus* and *E. coli*, respectively). When in direct contact with cells, inorganic NPs tend to act against pathogens through electrostatic attraction, ligand–receptor interactions, hydrophobic reactions, and van der Waals forces. Bioactive metallic ions are likewise released through the metal oxides that absorb the cell’s peripheral layers, allowing them to interact with the functional groups of cellular content such as proteins and nucleic acids, extra- or intracellularly. This triggers cell metabolic and structural changes, generating homeostatic imbalances [17]. All of this is facilitated if ZnO NPs are bioavailable, ergo peeking at the surface of PP and/or PA fibres.

The antiviral-related results followed a distinct pattern, with total eradication of the viral cells (according to the culture conditions tested) regardless of the functionalisation method used (**** *p* < 0.0001). The bacteriophage MS2 is a non-pathogenic (to eukaryote), icosahedral capsid, positive-sense single-stranded RNA virus and, thus, a potential surrogate of SARS-CoV-2. This way, the antiviral activity can be assessed safely and extrapolated to a real-life scenario [62]. Samples were examined for their ability in preventing viral droplets to permeate and infect agar plates and for their capacity to kill the MS2 virus upon incubation for 24 h.

The functionalised NWF2 by the dip-pad-dry methodology was shown to present the best antimicrobial properties among the ones tested. The lowest log values were obtained.

### 3.3. Facemask Characterisation

#### 3.3.1. Thermoforming Process

Thermal properties of NWF functionalised through the three distinct methodologies and using two concentrations (0.3 and 1.2wt%) of ZnO NPs loading were analysed by DSC and TGA, specifically evaluating the effect of the ZnO NPs incorporation, as well as the polymeric binder used for each methodology on thermal degradation behaviour.

**Figure 6:**
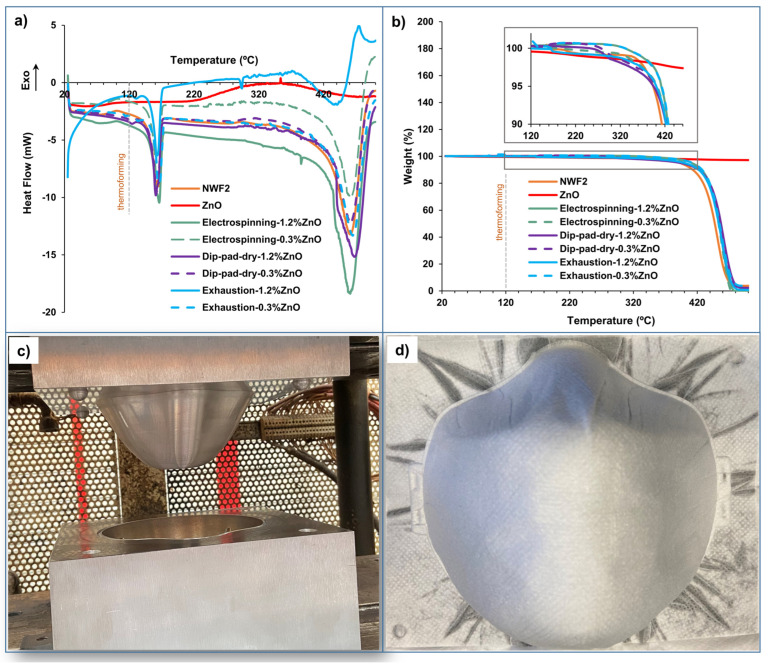
Thermoforming process: thermal evaluation of the control and functionalised samples through (**a**) DSC thermograms obtained at a heating rate of 10 °C/min, from 25 to 500 °C; (**b**) TGA curves acquired at the same heating rate and temperature range (**a**); (**c**) set-up used for thermoforming process; and (**d**) thermoformed facemask.

The study of the thermal degradation profiles of the NWF2, before and after functionalisation (Figure 6a,b), enabled the determination of the ideal temperature to use in the pressing apparatus (Figure 6c) to assemble the three-layered fibrous structures into a facemask (Figure 6d). The ZnO NPs curve shows that this material is quite stable, not presenting a thermal degradation event until 500 °C, from both DSC and TGA results. In what concerns the control NWF2, the DSC thermogram exhibited a phase transition temperature at 120 °C and degradation of lateral chains near 160 °C, a fact that has also been reported elsewhere for other PP NWFs [63,64]). A large peak was detected at 450 °C, which is in accordance with the results obtained from the TGA analysis, being related to the decomposition of the main polymeric chains, leaving only carbon char [65]. The sample functionalised with 1.2wt% ZnO by exhaustion showed a different behaviour from the others, degrading at lower temperatures (400 °C), indicating that the functionalisation process negatively affected its thermal stability.

DSC thermograms of the functionalised PP NWF are shown in Figure 5a. PP NWF alone shows the main melting transition at 161 °C with the heat of melting of 82.62 J/g. The DSC curves of the electrospinning samples exhibited a main melting transition at 166.2 and 166.3 °C with the heat of melting of 87.33 and 102.52 J/g; for dip-pad-dry samples the main melting transition at 160.7 and 161.2 °C with the heat of melting of 87.49 and 79.71 J/g; and exhaustion samples the main melting transition at 163.4 and 164.1 °C with a heat of melting of 69.01 and 78.91 J/g. In this sense, all functionalised samples exhibited good thermal stability above the thermoforming temperature used to produce facemasks. On another hand, TGA curves are shown in Figure 5b. All functionalised samples presented a single-step thermal degradation profile in the temperature region from 330 to 500 °C, being the maximal degradation rate registered at 463.8 ± 3.0 °C (Table S1), which is mostly related to the degradation of PP fibres that constitutes the NWF. PP thermal degradation takes place in the temperature range from 300 to 493 °C with the melting step occurring at the temperature of 167 °C, as reported elsewhere [66,67]. As expected, all NWF samples presented an endothermic degradation profile with a mass loss of around 100% on the TG curve (Figure 5b): 96% for NWF2; 100% for electrospinning-1.2%ZnO and electrospinning-0.3%ZnO; 98% for dip-pad-dry-1.2%ZnO and dip-pad-dry-0.3%ZnO; 99% for exhaustion-1.2%ZnO; and 96% for exhaustion-0.3%ZnO. While specifically relating to the electrospinning-based functionalisation, PA was used as a binder of the active agent to the textile. The polymer exhibits a one-step degradation process, with a maximum temperature (Tmax) of around 450 °C [68,69]. Water content started to evaporate in the range of 50–120 °C, whereas thermal degradation could be observed between the temperature range of 350−500 °C, accordingly with Dabrowski et al. [70]. Through dip-pad-dry methodology, samples included PVA as binder, which traditionally presents three weight loss regions. The first region between 50 and 200 °C can be attributed to the loss of the absorbed water molecules, while the second one between 200 and 340 °C is related to the loss of water bound to the polymer matrix. The third region between 340 and 450 °C is associated with the decomposition and carbonization of the polymer [71]. Nevertheless, samples functionalised by dip-pad-dry did not evidence this characteristic thermal degradation profile, probably because these weight loss regions were masked by the PP degradation. Lately, the exhaustion functionalisation was performed using PEG as binder, which provided a lower degradation temperature, being Tmax around 386 °C [72]. However, this effect was not evidenced in the TGA curves from samples functionalised by exhaustion, once again due to the great contribution of the PP degradation on the weight loss registered. TGA profiles of the ZnO NPs revealed a total weight loss of 2.79%, mostly attributed to moisture content [73]. Nevertheless, as expected, no decomposition was detected, only for temperatures higher than 700 °C, accordingly with Shamsuzzaman et al. [74].

In summary, it is important to highlight that the selected temperature for the thermoforming process did not compromise the fibrous structure in either of the studied samples, as pinpointed by the grey dashed line of Figure 5a,b.

#### 3.3.2. Air Permeability

**Figure 7:**
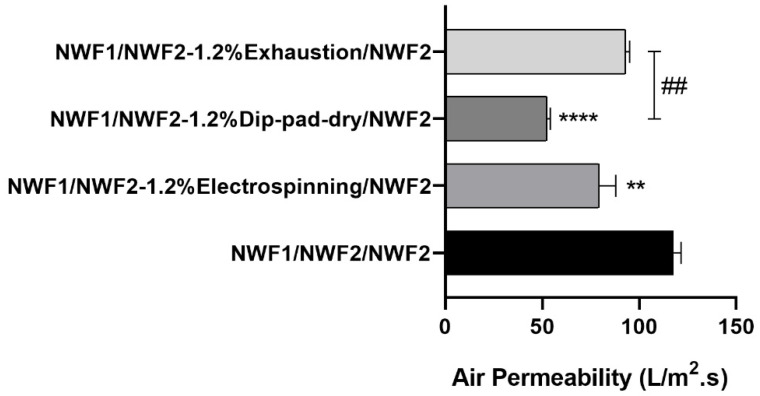
Air permeability assays with thermoformed three-layered system of control and functionalised samples. Statistically significant differences between functionalised NWF2, ## *p* < 0.01; and compared with the respective positive control, ** *p* < 0.01 and **** *p* < 0.0001.

The three-layered system has been optimized to function as an air filtration system, to promote micro- and NPs retention in respiratory masks. Air permeability was therefore evaluated to provide hints on this potential capacity (Figure 7). The functionalised filtering layer was combined with the other two selected layers, as depicted in the schematic Figure 1. Functionalized NWF2 exhibited a significant reduction of air permeability values in comparison with the control, with the dip-pad-dry methodology highlighting higher air permeability reduction (**** *p* < 0.0001). Moreover, in comparison with the exhaustion method, the air permeability of the facemask comprising dip-pad-dry was substantially lower (## *p* < 0.01). This was expected, in what concerns change induced by the electrospinning [5,7], dip-pad-dry [75,76] and exhaustion [77] functionalisation methods. Consequently, more opportunities are created for harmful particle collision with the fibrous structures following their contact with the multilayer [5].

#### 3.3.3. Breathability and Particle Retention

Following analysis of air permeability values, facemask breathability and particle retention capacity were studied according to the BS EN 149:2001 + A1:2009 standard in order to validate the possibility of the proposed filtering system actually acting as a facemask [44]. In more detail, filtering masks of the type filtering face piece (FFP) are classified into three classes: FFP1, FFP2 and FFP3. The FFP1 is the least effective with 20% solid and liquid particle penetration while the FFP3 is the most effective, with only 1% of the particles being able to penetrate the filter. While analysing the particle retention capacity (Table 2), it is evident that NWF1 and NWF2 alone already fall within the FFP2 category, and that all three functionalisation methods turn the proposed facemask into a suitable FFP3 protective piece. Yet a facemask needs to allow its user to breathe without difficulties. Hence, in the inhalation step, with air passing at 95 L/min, the maximum permitted resistance is 2.1 (FFP1), 2.4 (FFP2) and 3.0 (FFP3) mbar, whereas to exhale, with air flowing at 160 L/min, 3 mbar is the allowed upper threshold. Joyfully, the breathability-related data of the produced facemask is lower than all of the defined limits. Water vapour permeability, WVP, was additionally examined to substantiate the above-cited breathability results, showing that the distinct functionalisation methods presented neglectable variations in comparison with the control: control (I = 78.77%), NWF1/NWF2-1.2%exhaustion/NWF2 (80.82%), NWF1/NWF2-1.2%electrospinning/NWF2 (77.40%).

#### 3.3.4. Biological Studies

**Figure 8:**
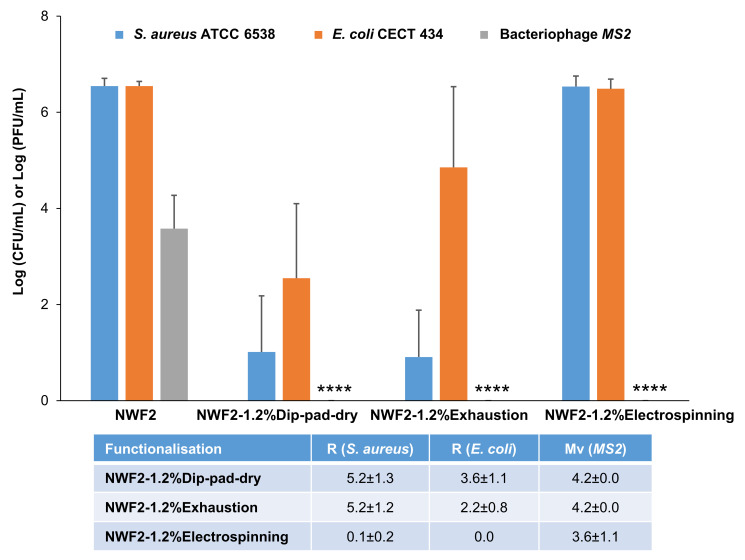
Antimicrobial activity of thermoformed NWF2 and functionalised samples through three methodologies against *Staphylococcus aureus* ATCC 6538, *Escherichia coli* CECT 434 and bacteriophage MS2, and their respective antibacterial activity (R) and antiviral activity (Mv) values. **** *p* < 0.0001 compared with the respective positive control (without functionalisation).

Finally, conclusive data regarding the capacity of the facemask to provide the expected biological protection and additional biological studies were conducted. The goal was to show that the filtering layer can eliminate the needed biological threats. For that, the facemask was built, with the middle layer being then extracted and evaluated (Figure 8). The obtained results confirm what was previously obtained for the cultured filtering layer before thermoformation. All three protocols under study lead to total viral removal from the textile surface after incubation (**** *p* < 0.0001) and dip-pad-dry presented the most effective antibacterial activity. Thus, the results obtained showed that NWF functionalised through the dip-pad-dry methodology exhibited the most attractive antimicrobial ability.

## 4. Conclusions

In summary, three distinct methods of functionalisation of NWF with ZnO NPs were exploited and characterized. The dip-pad-dry functionalisation methodology provided good antibacterial properties and excellent antiviral characteristics, thereby provoking substantial (** *p* < 0.01 for *S. aureus* or **** *p* < 0.0001 for *E. coli*) and total (**** *p* < 0.0001) cell death, respectively. Since this was achieved using low amounts of active agent, the present results confirmed the high potential of ZnO NPs as an efficient neutralising agent for active antimicrobial textile finishing. Moreover, three-layered NWF-based systems were assembled by thermoforming to obtain facemask prototypes that were further characterized. Facemasks incorporating functionalised NWF through the dip-pad-dry methodology as a filtering layer exhibited optimal permeability (50 L/m^2^·s), breathability (0.175 mbar for inhalation and 0.531 for exhalation steps) and particle retention capabilities (0.03%), and so are tailored to meet the specific requirements of their intended application. Moreover, these masks retained the essential antimicrobial properties offered by the active filtering layer embedded with ZnO NPs. The innovative three-layered active filtration system, as proposed in this work, emerges as a highly promising solution, enabling the creation of enduring and efficient facemasks, ultimately contributing to a more sustainable approach in healthcare and various other sectors.

## Figures and Tables

**Table 1:** Characterisation of the NWFs.

Studied Parameter	Result Output	NWF1	NWF2
Structural	Thickness (mm)	0.76 ± 0.03	0.55 ± 0.02
	Areal mass density (g/m^2^)	92.46 ± 3.41	56.02 ± 1.74
Physical	Water contact angle (°)	125.36 ± 11.91	127.84 ± 5.47
Mechanical	Bending stiffness (mg.cm)	3184.99 ± 0.29	708.69 ± 0.25
	Static µ	0.25 ± 0.018	0.27 ± 0.023
	Kinetic µ	0.19 ± 0.009	0.21 ± 0.006
Thermal and moisture management	One-way transport capacity, OWTC (%)	862.35 ± 42.02	881.43 ± 126.58
	Overall moisture management capacity, OMMC	0.67 ± 0.02	0.73 ± 0.09
	Thermal conductivity λ (10^−3^ W/m K)	31.27 ± 0.31	28.83 ± 0.40
	Thermal diffusion α (10^−6^ m^2^/s)	0.126 ± 0.021	0.179 ± 0.025
	Thermal absorptivity b (Ws^1/2^/m K)	89 ± 8	69 ± 6
	Thermal resistance R (10^−3^ m^2^ K/W)	24.17 ± 0.97	19.10 ± 0.70
	Thermal diffusion P (10^−6^ m^2^/s)	2.08 ± 0.13	1.49 ± 0.06

**Table 2:** Breathability and filtration capacity of NWF1, NWF2, and three facemasks thermoformed following functionalisation by electrospinning, exhaustion and dip-pad-dry.

	NWF1	NWF2	NWF1- NWF2(dip-pad-dry)-NWF2	NWF1- NWF2(exhaustion)- NWF2	NWF1- NWF2(electrospinning)-NWF2
Pressure_Inhalation_ (mbar)	0.679	1.104	0.175	1.935	1.992
Pressure_Exhalation_ (mbar)	0.879	0.864	0.531	1.307	1.226
Particles penetration (%)	4.142	4.479	0.030	0.000	0.073

## Data Availability

The data presented in this study are available on request from the corresponding author.

## Supplementary Materials

The following supporting information can be downloaded at: https://www.mdpi.com/article/10.3390/polym15173499/s1, Figure S1: TEM characterisation of ZnO NPs: (a) Representative image; and (b) size distribution and average diameter; Figure S2: Calibration curve of ZnO NPs obtained from UV-Vis spectrophotometry based on the absorbance values registered at 365 nm with increasing concentration of the ZnO NPs aqueous suspensions.; Figure S3: EDS spectra obtained for distinct NWF substrates: (a) NWF2; (b) Electrospinning-1.2%ZnO; (c) Dip-pad-dry-1.2%ZnO; and (d) Exhaustion-1.2%ZnO.; Table S1: Thermal degradation parameters determined from the experimental TGA curves..
