# Supplementary material for: Comparison of Zinc Oxide Nanoparticle Integration into Non-Woven Fabrics Using Different Functionalisation Methods for Prospective Application as Active Facemasks
Source: Polymers (Basel). 2023 Aug 22;15(17):3499. doi: 10.3390/polym15173499 (PMC10489795; doi:10.3390/polym15173499)
Supplement: Supplementary file 1 [file polymers-15-03499-s001.zip › polymers-2521522-supplementary.pdf]

Supplementary Files

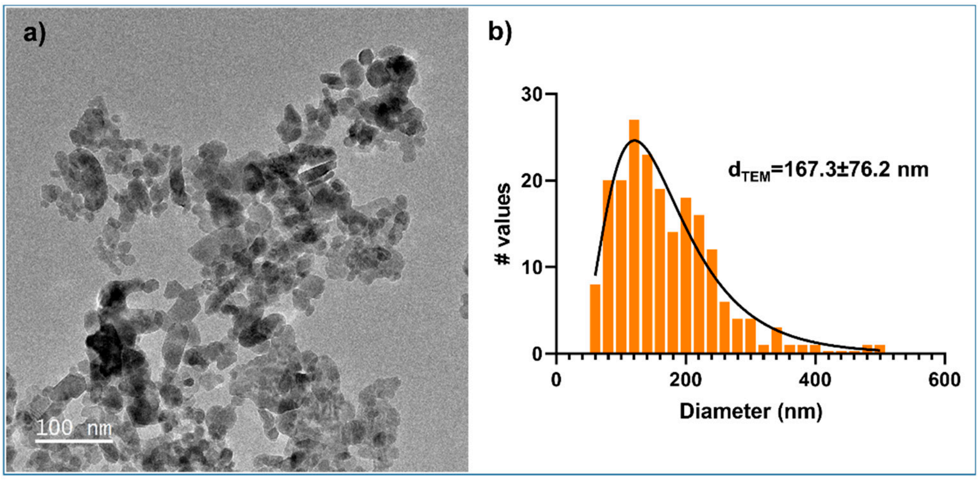

Figure S1. TEM characterisation of ZnO NPs: a) Representative image; and b) size distribution and average diameter.

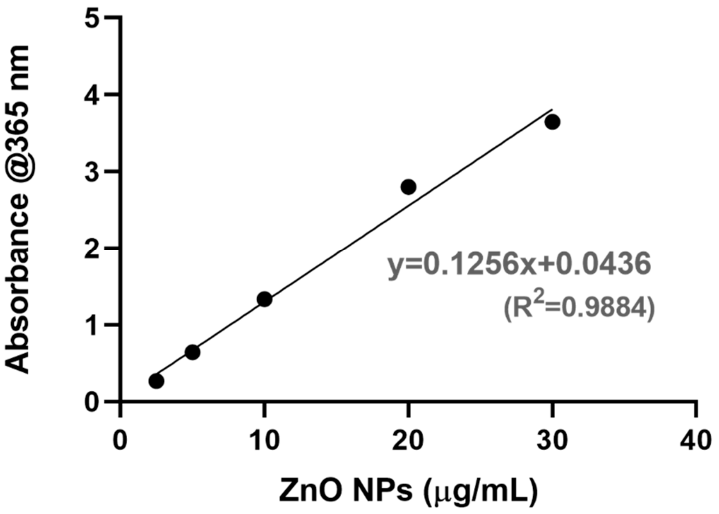

Figure S2. Calibration curve of ZnO NPs obtained from UV-Vis spectrophotometry based on the absorbance values registered at 365 nm with increasing concentration of the ZnO NPs aqueous suspensions.

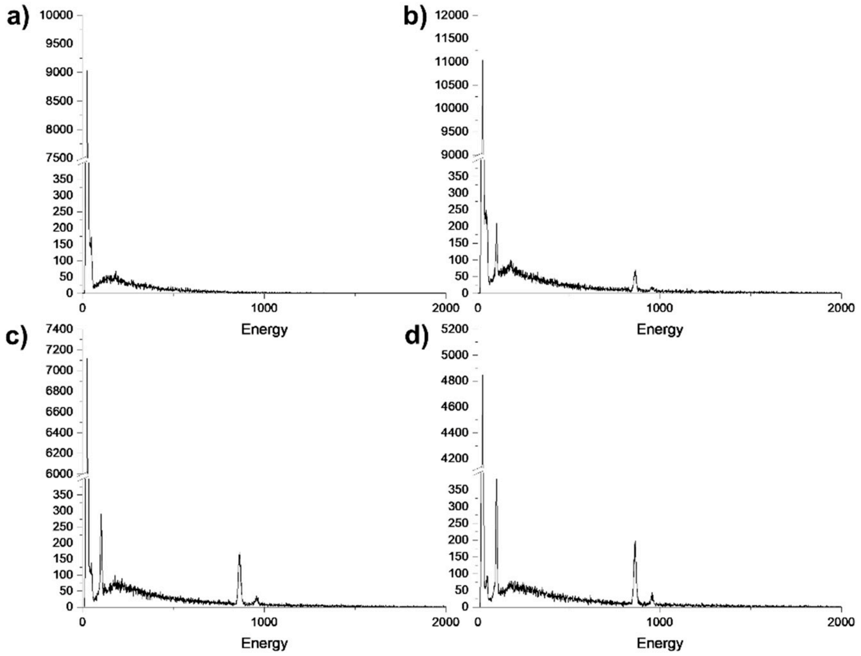

Figure S3. EDS spectra obtained for distinct NWF substrates: a) NWF2; b) Electrospinning-1.2%ZnO; c) Dip-pad-dry-1.2%ZnO; and d) Exhaustion-1.2%ZnO.

Table S1. Thermal degradation parameters determined from the experimental TGA curves.

Samples	$T_{10\%}$ (ºC)	T_{peak} (ºC)	Mass residue at 500ºC (%)
NWF2 (control)	413.103	461.36	3.854
Electrospinning-1.2%ZnO	426.8	460.98	0
Electrospinning-0.3%ZnO	422.23	463.15	0
Dip-pad-dry-1.2%ZnO	421.89	468.56	2.37
Dip-pad-dry-0.3%ZnO	422.97	461.27	1.70
Exhaustion-1.2%ZnO	422.06	462.80	0.83
Exhaustion-0.3%ZnO	427.37	466.19	3.88
